# Forensic trace DNA: a review

**DOI:** 10.1186/2041-2223-1-14

**Published:** 2010-12-01

**Authors:** Roland AH van Oorschot, Kaye N Ballantyne, R John Mitchell

**Affiliations:** 1 Forensic Services Department, Victoria Police, 31 Forensic Drive, Macleod 3085, Victoria, Australia; 2 Department of Forensic Molecular Biology, Erasmus University Medical Center, 3000 CA Rotterdam, The Netherlands; 3 Department of Genetics, La Trobe University, Melbourne, Victoria 3086, Australia

## Abstract

DNA analysis is frequently used to acquire information from biological material to aid enquiries associated with criminal offences, disaster victim identification and missing persons investigations. As the relevance and value of DNA profiling to forensic investigations has increased, so too has the desire to generate this information from smaller amounts of DNA. Trace DNA samples may be defined as any sample which falls below recommended thresholds at any stage of the analysis, from sample detection through to profile interpretation, and can not be defined by a precise picogram amount. Here we review aspects associated with the collection, DNA extraction, amplification, profiling and interpretation of trace DNA samples. Contamination and transfer issues are also briefly discussed within the context of trace DNA analysis. Whilst several methodological changes have facilitated profiling from trace samples in recent years it is also clear that many opportunities exist for further improvements.

## Aims and scope

Trace DNA analysis has become an integral part of a forensic laboratory's workload and a key tool for investigators. Accordingly, there has been considerable research conducted in order to investigate the characteristics of trace DNA and the best methods to improve its collection, amplification and interpretation. This review aims to provide a brief history of trace DNA and to summarize some of the methods and techniques used to collect, amplify and interpret the smallest samples encountered in forensic biology. Although it is not possible to provide an exhaustive description of all minutiae, or all of the numerous cases where trace DNA has provided key evidence, we hope that the reader will gain an appreciation of the complex nature of trace DNA, the current state of knowledge and the areas where research remains to be done.

## Brief history

### Brief history of polymerase chain reaction (PCR)-based DNA profiling in forensic casework

In the early 1990 s, forensic science started moving away from markers such as D1S80, consisting of large core repeat units and overall large amplicon size [1-4] to short tandem repeats (STRs) [5-12]. The first widely used commercial kits designed for the typing of multiple STRs in a single reaction became available in the late 1990s/early 2000s [13-18]. PCR allowed the generation of genetic information from minute amounts of DNA; multiplexing of primers allowed the generation of genetic data from multiple sites from the same aliquot of DNA thus reducing sample consumption; fluorescent primers assisted multiplexing and new automated typing systems; and the use of STRs improved the chances of profiling poor quality samples. As the desire for higher discrimination power between individuals arose, the number of loci targeted by a single multiplex increased and there are now a number of commercially available, well-validated kits, incorporating 15 - 16 highly variable STR loci (plus amelogenin), such as PowerPlex^® ^ESX and ESI systems [19-21] and AmpF*l*STR^® ^NGM [22]. These new kits also include improvements in primer design, buffer composition and amplification conditions which improve the analysis of trace samples [19-22].

The desire to compare DNA profiles between cases over time and across jurisdictions (and the associated legislations in many countries around the world supporting the establishment of National DNA databases) dictated that the forensic community select a core set of loci for the generation of DNA profiles. The international forensic community identified a small set of core loci mainly consisting of tetranucleotide tandem repeat loci [23,24]. Although the loci used by each jurisdiction are not always the same, there is a considerable amount of overlap in the loci used among the jurisdictions.

Although the establishment of a consensus set of core STR loci allows comparisons of profiles across jurisdictions and over time through use of national databases, it may also be simultaneously stifling opportunities for the improvement in the quality and efficiency of the service provided. Changing the type of markers used, from STRs to single nucleotide polymorphisms (SNPs), may result in increased success from more forensic samples and be more adaptable to high-throughput and/or miniaturized typing systems. Theoretically, the smaller amplicon sizes of SNPs lend themselves well to the production of genetic profiles from both degraded and trace DNA. Their reduced level of polymorphism relative to the routinely used STRs is, however, a disadvantage. With sufficient numbers this can be overcome, although it may make mixture resolution more difficult. Whilst sensitive SNP-based individualization profiling systems are available [25,26] they are not routinely used.

A major improvement that the forensic community has developed to assist in the typing of degraded and trace DNA has been the recent re-engineering of primers of the core STR systems, such that they are positioned closer to the repeat units in order to reduce the length of the amplified flanking regions [27-36]. The use of mobility-shifters (non-nucleotide linkers, Applied Biosystems, CA, USA ) and novel multiplex design strategies [such as Promega's ESI and ESX kits (WI, USA)] have allowed all the core CODIS (Combined DNA Index Systems) and European set of loci to be amplified as 'miniSTR' loci, thus increasing the profiling success rate from difficult samples [19,35,37].

### Brief history of biological sample types collected and typed for forensic investigations

PCR-based methodologies allowed for the generation of profiles from types of samples not previously examined - such as cigarette butts [38], single human hairs [39], urine [40], fingernail scrapings [41] and bite marks [42] - and also improved the success in generating useful profiles from old, burnt, degraded bone and tissue samples [34,43-50].

In 1997 it was reported that DNA profiles could be generated from touched objects [51]. This opened up possibilities and led to the collection of DNA from a wider range of exhibits (including: tools, clothing knives, vehicles, firearms, food, bedding, condoms, lip cosmetics, wallets, jewellery, glass, skin, paper, cables, windows, doors and stones) [52-69]. A concomitant increase in the application of DNA profiling in investigations of a wider range of offences, including theft, homicide, clandestine laboratories, armed robbery, assaults, sex offences and volume crime, accompanied the increase in types of exhibits. The ready availability of DNA from touched objects has also assisted in the success of many national offender DNA databases [70-76] as many focused on including profiles from perpetrators of volume crimes (such as burglary, vehicle crimes, street robbery and drug cases). The traditional forensically relevant biological substances, such as blood and semen, are not commonly encountered at these sorts of crime scenes but touched objects provide a wide scope for revealing the offender's DNA profile, and the frequency of recidivism in these crimes increases the probability of multiple hits across databases.

In the early years of trace DNA profiling there were several sceptics within the forensic community about the possibility of obtaining DNA profiles from touched objects. However, after conducting their own research they revised their views on trace DNA. In addition to the sceptics, there were forensic laboratory managers who disliked the potential difficulties trace DNA brought, as they foresaw a significant increase in sample submissions which they did not have the necessary resources to process. However, many later used the favourable prospect of trace DNA to argue for increased staff and budgets and improved laboratory facilities. The later application of methodologies designed to increase the likelihood of obtaining useful profiles, specifically from very minute samples such as the low copy number (LCN) methodology with extra cycles or low template DNA (LTDNA) methods, have further increased the opportunity to generate profiles from trace crime scene samples [77-81]. However, these applications can bring their own challenges [82], which have reignited the debate over the use of trace DNA in the forensic field [83-87].

What is currently commonly referred to as 'trace DNA' or 'touch DNA' has previously been referred to as low copy [77,88], or low template [82,89]. In our view, the application of a single term, such as trace DNA or LTDNA, can be a misleading simplification of a series of complex processes. Trace or touch DNA may be the appropriate term when referring to the collection of minute biological samples at the crime scene or the process of collecting and extracting the tiny amounts of material within the sample in the forensic laboratory. Low template is used as a descriptor for the amplification phase, where the use of low amounts of material is likely to generate stochastic effects. While the term LCN also relates to low template, it tends to be used to describe the process of increased cycle number rather than the amount present. The profile could equally be referred to as 'low level' in the interpretation phase, reflecting that the peak heights are below a validated threshold level. For consistency, within this review we will use the term 'trace DNA' to refer to any sample which may fall below the recommended thresholds at any stage of the process - detection, collection, extraction, amplification and interpretation.

Trace DNA typically refers to either the very limited and/or invisible biological samples and/or amounts of DNA less than 100 pg [88]. However, some laboratories use a 200 pg limit as the threshold limit [82,90]. Recently, there has been some discussion about eliminating template thresholds entirely from the definition, as they represent an artificial cut-off for a phenomenon which is continuous. Instead, a risk assessment based on peak heights of the resultant DNA profile can be used to determine whether or not an appropriate amount of DNA was present and whether or not stochastic factors affected the genotype result [91]. It should be recognized that an apparent trace DNA sample at a particular processing phase does not necessarily mean that it, or the profiles generated from it, will continue to be considered a trace DNA sample in subsequent phases. It should also be understood that, as methods change, any defined threshold amount for trace DNA will also probably change. We encourage any biologists working with trace DNA amounts to consider all aspects of the process, rather than simply focussing on the interpretation phase. Working effectively with trace DNA necessitates an understanding of factors relating to its collection, extraction, amplification and interpretation, as well as issues relating to contamination and transfer.

## Trace sample collection

### Targeting

The first step in collecting trace samples is to identify which areas to target. By and large, trace samples on surfaces are not readily identifiable. Whilst finger printing agents are used to identify touched areas on some exhibits, many exhibits are swabbed or tape lifted based on assumptions about where the DNA-containing material is located. The use of non-invasive detection systems would be ideal. One such system is the application of light sources, such as the Polilight [92-97], but the use of these is not as widespread as it could be. This may be due to a lack of awareness of their usefulness, or their perceived impracticality, or their non-ideal performance for specific tasks or that further investigative and validation work is required to define their scope and limitations. Touched surfaces that have been revealed using fingerprinting methodologies are usually those surfaces on which fingerprints are sought as the priority, rather than surfaces where DNA will be sampled. Although many fingerprinting methods do not adversely affect the quality of retrieved DNA, some methodologies may do so [52,66,98-104] and others may reduce the quantity of retrieved DNA [66,69,105]. When considering the downstream use of the fingerprint, more emphasis should be given to the impact of the fingerprinting methodology used on subsequent DNA retrieval and quality.

Improvements in the methodologies for identifying the biological source of trace samples (not just fingerprints) on exhibit surfaces, and their application in the course of forensic investigations, should help to improve sample collection. Swabbing an assumed trace sample area that is smaller than the actual deposition area will mean that some of the sample goes uncollected. Swabbing an area greater than the actual area of deposit may mean that sample is spread over a wider area and that less is collected. Both approaches also have the potential to give an inaccurate view of where the actual sample was located. It is, therefore, not only necessary to be aware of the precise location of the material being targeted but also to collect from the area appropriately.

### Collecting methodologies

Most trace samples are collected using swabs. Swabbing an area requires a moistened swab to traverse the whole target area multiple times with some pressure and rotation of the swab so that the full surface area of the swab can contribute to the collection. However, a moist cotton swab does not pick up all of the available biological material from the surface and, in many instances, it may pick up less than half the available sample [106,107]. Swabbing an area with multiple swabs, and the co-extraction of these swabs, has been advocated in order to enhance overall retrieval of DNA [65,105,108,109]. It is now common practice to perform a double swabbing technique. Some moisten the first swab but leave the second swab dry. Keeping the second swab dry, if the first one has already absorbed all the moisture it contributed to the sample, may not collect as much of the target as if it too was moistened prior to swabbing. In this case it is important to ensure that all its moisture is recaptured.

Although water is mainly used as the moistening agent [57,107], some laboratories use 0.01% sodium dodecyl sulphate [110] or isopropanol (MiniPopules, PuritanMedical, Maine, USA) [111]. Limited research has been dedicated towards identifying an optimal solution for the retrieval of DNA from touched objects. It is possible that an empirical study of agents, or a combination of agents, with both dissolving and abrasive properties will reveal improvements. Some surfaces are more difficult to collect samples from than others [57] and the use of different moistening agents for different surfaces may facilitate collection.

Various types of cotton swabs are used but other swab types such as foam are also in use [57,111,112]. The type used for trace DNA collection can be a matter of convenience (already used for the collection of other sample types) or price. Few practitioners have undertaken studies to check if the swab type used is better or worse than the alternatives for the purpose of collecting touch/trace samples. The identification of the optimal collection methods and swab types for particular samples on particular substrates would be beneficial. It is also important that the swabs be DNA-free (and not just sterile), in order to avoid the problems seen in the recent 'Phantom of Heilbronn' case discussed and referred to below.

Once the DNA-containing material has been collected, most methodologies require the DNA to be extracted from the collection device. In the case of a cotton swab, it has been shown that some commonly used DNA extraction methodologies are not particularly efficient in retrieving all the DNA collected from the swab [105]. It may be easier to retrieve DNA from some swab types than others and particular DNA extraction methods and protocols may, in turn, be better than others. A comprehensive comparison of extraction methodologies/protocols from various collection devices used specifically to collect and retrieve touch/trace samples, will assist investigators in developing and applying the most successful protocols.

If a swab used to collect biological material is allowed to dry prior to extraction less DNA is retrieved than if the still moist swab was processed immediately [105]. Freezing the collection swab rather than drying it prior to extraction results in DNA recovery rates approaching that of a moist swab [113]. In some laboratories it is now common practice to freeze collection swabs immediately after collection.

Early on, it became apparent that swabs of trace samples found on clothing did not provide high-quality results as could be expected. The swabs frequently contained inhibitory factors and/or provided profiles that were difficult to interpret. Tape is now used regularly to retrieve DNA containing material from worn clothing [57,112,114]. By pressing a strip of tape multiple times over the target area, the most recently deposited material, with fewer inhibitory factors, are collected and accumulatively they provide better profiles [115,116]. Some laboratories are using tape or sticky tabs not only to collect biological material from clothing, but also from other touched surfaces. However, taping of larger surfaces can be tedious and the multiple strips of tape that are required can pose downstream processing difficulties. Franco and Goetz [115], Jiang [117] and Berschick [118] have all demonstrated that the use of filtered tips and vacuums could be useful alternatives for collecting biological material, particularly from large surface areas.

The above collection methods do not distinguish between the contributing sources of DNA-containing material. The desired target DNA is often a trace component of a large amount of DNA-containing material that emanates from multiple sources. The use of laser microdissection techniques allows sufficient numbers of relevant target cells to be isolated from the other overwhelming cell types. Microscopically, different cell types can be distinguished based on morphological characteristics, various chemical staining or fluorescence labelling techniques. Using this technology a clear DNA profile derived from the minor cell type alone can be generated that otherwise would have either been swamped by the major component and not been detectable in the profile, or have been part of a complex and more difficult profile to interpret [119-128]. Anslinger *et al. *[125] and Vandewoestyne *et al. *[128] have demonstrated that, even when the cell type of two contributors in a mixture is the same, the cells derived from a male contributor and a female contributor can be distinguished from each other using fluorescent probes and separated accordingly using the laser microdissection methodology. Although laser microdissection has been shown to be exceptionally useful for separating contributors, the preparation of the sample may act as a limitation to its widespread forensic use, as coated glass slides are required. A sample must be transferred from the collection device to the slide, which may result in the loss of cells. It would be preferable to use laser microdissection methodology directly on the initial collection device. Alternatively, the development and application of appropriately sticky flexible slides or tapes for direct collection could increase the application of laser microdissection. Furthermore, the identification of relevant cell types is currently often done manually and is time consuming. Further developments of automated cell type recognition software would be welcome.

Another approach to separating contributors in sample mixtures is the application of flow cytometry/cell sorting methodologies that are regularly used in some non-forensic applications [129,130]. Single cell sorting has been routine in many fields for years and allows the specific separation of heterogeneous mixtures of cell types [131]. Newer cell sorting technologies, such as the Becton-Dickinson NJ, USA) FACSAria III, allow the simultaneous separation of up to four distinct populations of cells, based either on the morphology of the cell or the use of specific antibodies against each cell type. Single or multiple cells can be sorted directly into PCR tubes or onto glass slides for low volume PCR applications. Although the cell sorting methods are faster and more easily automated than laser microdissection, the often degraded nature of forensic samples may create difficulties for this method which requires that the cells be largely intact and within a fluid matrix [132]. However, flow cytometry has previously been successfully applied to separate sperm cells from vaginal wash fluid following intercourse [132-134]. Provided technical difficulties can be overcome, flow cytometry and cell sorting may provide a successful method for the separation of complex mixtures of small numbers of cells.

Whilst swabbing and taping a touched area for retrieval of DNA seems simple and straightforward, training exercises with would-be collectors have demonstrated how easy it is to get wrong. Inadequate training, combined with the absence of competency testing and ongoing monitoring of an individual's techniques, could drastically limit the success rates. There is a clear need for improved initial and ongoing training, proficiency testing, success-rate data collection and bench marking [135,136].

## Trace DNA extraction

For many years forensic practitioners have relied on Chelex 100 (Bio-Rad, CA, USA) [137] and organic methods [138] to extract DNA from their samples. Commercially available kits and methodologies, optimized for specific types of samples, became prominent during the 1990s [139,140]. The last few years have seen developments which utilize silica-coated magnetic beads to capture DNA from the rest of the lysed cell [such as Promega's DNA IQ (WI, USA) and Invitrogen's Chargeswitch (CA, USA)] and optimization of these to suit robotic systems [141,142].

Several DNA extraction methods utilized by forensic scientists do not recover all of the collected DNA, with losses of up to 75% occurring from Chelex and organic extraction methods [105,142,143]. Some of the loss is influenced by the substrate on which the sample is presented but the majority is due to the methodology. The bead extraction systems tend to have binding thresholds (well above trace amounts) over which the remaining DNA is discarded. The majority of samples submitted for profiling contain relatively large amounts of DNA well above the 0.1-0.5 ng minimum required by most common STR profiling systems. Below this amount standard methodologies tend to provide mainly partial profiles (see below). Accordingly, it is not relevant in many cases that there is some loss of DNA during the extraction process. Few studies have, however, focussed on optimizing extraction techniques specifically for trace DNA samples.

Most extraction methods result in DNA in a relatively large volume. There may be some jurisdictions that require the retention of a specific portion of the collected sample for repeat analysis. However, when this is not the case, and when dealing with a trace sample, the elution of the DNA in a relatively large volume could be limiting. Whilst there may be sufficient DNA in total to acquire a good profile, the concentration may be so low from trace samples that when only a proportion of the available volume is utilized in the amplification, less than optimal profiles are generated. Concentration and clean-up devices, such as Microcon (Millipore, MA, USA), MinElute (Qiagen, CA, USA) or NucleoSpin (Clontech, CA, USA) DNA clean-up columns, are used to help concentrate and/or clean-up samples prior to amplification [144,145]. However, some of the benefit of their use is negated by the loss of some DNA that these methods entail [105]. This loss could potentially be minimized by the addition of Poly A RNA or salmon sperm DNA to the concentration device [144].

As most extraction methods have a relatively low efficiency rate, it would be desirable to optimize methodologies specifically for trace samples so that one has the opportunity to: (a) extract most of, if not all, the available DNA; (b) remove all amplification inhibiting elements without the loss of DNA; (c) utilize all of the extracted DNA for amplification; and (d) add the amplification reagents to the vessel containing the DNA rather than having to transfer the DNA to a separate vessel containing the amplification reagents (thus avoiding a further loss that could be encountered in the transfer step - for example, retention of DNA on vessel walls and/or in pipette tips). The development of direct PCR from 1.2-2 mm FTA card punches containing saliva or blood, without the need for pre-washing or the removal of heme, is a step toward the latter [146,147]. However, at present, the direct PCR methods are not yet able to amplify swabs or tape-lifts which would be required with trace DNA.

## Trace DNA quantitation

Although it may not always seem necessary to quantitate trace samples, given the expected low concentration of DNA, it can be helpful to have an indication of the approximate quantity present when interpreting the resultant STR profile. In addition, it is not always correct to assume that touched objects contain only low amounts of DNA. Depending on the nature, frequency and duration of the contact, tens or hundreds of nanograms may be present [51,65,66,148]. As such, quantification of every sample can ensure the maximum efficiency and prevent repeat analyses of over-amplified samples [82]. However, a negative quantitation result should not prevent the downstream processing of trace samples. Partial or even complete 9 locus STR profiles have been obtained from samples giving a negative result with the most commonly used forensic quantification kit, the Quantifiler Human DNA Quantification kit (Applied Biosystems, Foster City, CA, USA) [149]. Most probably, stochastic sampling and amplification effects are operating on the real-time quantitation method with extremely low template amounts and, as such, any quantitation result of very low amounts of template must be taken as an indication of the concentration, rather than as an absolute measurement as with higher input amounts.

It is a common practice in some laboratories to use only a portion of the extracted DNA for amplification due to; (a) internal methods, procedures and policies; (b) a lack of willingness to concentrate the sample for laboratory efficiency reasons; and/or (c) a desire to retain a certain portion of the sample for possible future further typing. A deeper consideration of workflow processes and priorities may yield an alternative protocol that will allow the use of a greater portion of the available DNA for amplification thus increasing the chance of generating fuller and easier to interpret profiles.

## Trace DNA amplification and detection

### General

In recent years, the most intensive research and discussion in the field of trace DNA has been in the areas of amplification and interpretation of low level template amounts. Perhaps this focus reflects the perceived ease of optimising this aspect, in comparison to the others discussed above, or that amplification is the main area where the forensic molecular biologists have control of the quality of the sample and analysis; the other areas being more the domain of the criminologist/crime scene officers, or indeed simple chance. Nonetheless, continued research has led to some significant improvements in the way forensic molecular biologists amplify and interpret trace DNA profiles but it has also raised many more questions and resulted in research areas opening up, particularly in the statistical interpretation of profiles.

### Improving amplification

The most commonly used method of enhancing the success of trace DNA amplification is to increase the number of cycles. This procedure, developed at the Forensic Science Service (FSS) in the UK (and adopted by other laboratories), is often referred to as an LCN analysis [77]. Over 21,000 samples have been analysed with this technique (in the UK alone) at the time of writing, representing a significant portion of the overall caseload [150]. For this LCN technique the number of cycles used during the PCR of the STR loci is increased to 34, potentially allowing an increase in product of over 3 billion, compared to the standard 28 cycle reaction. Increased cycle number is commonly used in research and ancient DNA settings, where up to 60 cycles may be used to maximize the success of the amplification [151].

Many papers have described the efficacy of increasing cycle numbers [77,78,152]. In the original description of LCN, complete profiles from only 5-10 cells (30-60 pg) and substantial increases in peak heights were reported [77]. Kloosterman and Kersbergen [152] used a slightly different strategy in which additional *Taq *polymerase was added after 28 cycles, and thermal cycling continued for a further six cycles. Single cells were reportedly analysed successfully, with as much as 6 months difference between the first 28 and second six cycles. Although most of the older STR profiling kits recommend only 28 cycles, new kits are taking advantage of the improved success resulting from the increased number of cycles, such as the Applied Biosystems (CA, USA) Minifiler (29 cycles), Yfiler (30), and NGM (29) and also Promega's ESX and ESI kits (30 cycles). This development will allow those laboratories that are reluctant or unable to perform the LCN methodology to benefit from the advantages of an increased cycle number. However, it also brings with it a need to increase the stringency of contamination prevention, as it is likely that more sporadic contamination will be detected due to the increased sensitivity.

Primer design for the amplification of the STR loci in the commercial multiplexes can be, and has been, improved. While the majority of kits still use the original primers, Butler *et al. *[28] redesigned the amplicons to produce 'mini-STRs', producing primers which had significantly higher success rates with degraded DNA due to the smaller amplicons. This concept of generating smaller amplicons was used to produce the MiniFiler STR kit (Applied Biosystems), which has a considerably higher success rate with degraded or inhibited DNA than the standard AB kits, but also has a lower template input requirement - 0.125 ng compared to 0.5 ng [35]. This decreased threshold may reflect the increased priming and amplification efficiency of the new primers, but also that the optimization of the multiplex can play a large part in the sensitivity of the amplification.

However, there is still more that could be done to improve the amplification success of forensically relevant STR loci. Previous research has shown that the incorporation of synthetic nucleotides with stronger binding capabilities than standard nucleotides into PCR primers can substantially improve the amplification success of low template levels. Locked nucleic acids, an RNA analogue, have a binding strength far superior to DNA, giving increased sensitivity and specificity [153,154]. Ballantyne *et al. *[155,156] have shown that incorporating a small number of locked nucleic acid (LNA) bases into the mini-STR primers can increase amplification success of trace DNA samples by over 300%, suggesting that the incorporation of LNA into primers should be examined as a tool to increase amplification of forensically relevant samples in new commercial multiplexes.

In addition to altering the cycling conditions or primer sequences, the master-mix components, and the molecular mechanisms by which they can interact, can be altered. Reducing the volume of the PCR can have the most substantial effect. Gaines *et al. *[157] reported four-fold increases in sensitivity when the volume of reaction was reduced to 5 μL, while using 1 μL on glass slides allowed the amplification of 15 STRs from only 32 pg of DNA [158]. However, simply reducing the volume of an amplification may not increase efficiency or accuracy, it may simply increase the relative concentration of the template relative to the reagents. Although this will not remove all stochastic effects of low template amounts, it can reduce their impact on the resultant STR profile.

Altering specific components (such as magnesium chloride, primer or buffer concentrations) within the commercial STR multiplexes is not possible, due to the way they are prepared. There have been some suggestions that altered buffer formulations or primer concentrations may improve the amplification success of low level samples [146,147], although these remain untested with the current STR multiplexes. The newer commercial multiplexes such as MiniFiler and NGM do, however, have optimized buffer systems to cater for inhibited samples, which may be a factor in their increased sensitivity. However, altering the type of DNA polymerase has been shown to substantially increase reaction sensitivity and efficiency, giving improved results with low template amount or inhibited casework samples [159]. In the same way that alternative DNA polymerases have been examined, it would be desirable to see empirical studies of the effects of alternative concentrations of other components such as magnesium chloride, dNTPs and primers at trace DNA levels.

Another alternative worth considering is the addition of chemical adjuvants to increase reaction efficiency or sensitivity. Bovine serum albumin (BSA) can prevent inhibitory substances reducing the activity of *Taq *polymerase by sequestering phenolic compounds which may otherwise scavenge the polymerase [160] and its presence may reveal sufficient amplifiable DNA in samples which otherwise appear to contain only trace amounts. Although BSA will not increase amplification sensitivity from high-quality, inhibitor free DNA samples, many forensic samples may contain low levels of inhibitory substances and, thus, most commercial assays contain a low percentage of BSA as a standard component.

Instead of increasing the amplification efficiency, the amount of template added to the PCR can be increased to facilitate increased STR profiling success. Whole genome amplification (WGA) can assist by replicating the entire DNA sample, rather than the specific target as with PCR, producing hundreds of nanograms from picogram input amounts. This increase in DNA gives the analyst the opportunity to add 10 or 100-fold more DNA to the genotyping PCR, substantially increasing the probability of obtaining a complete profile. Additionally, it allows multiple, high quantity PCRs to be performed and generates sufficient DNA for archiving purposes. The commercially available methods work optimally on 1-10 ng of DNA, although they have been successfully used on single or small numbers of cells in non-forensic areas [161-163], and generate sufficient DNA to analyse hundreds of genes. However, substantial amounts of amplification bias have been observed between homologous alleles and loci with low template amounts with WGA [164,165]. Despite this problem, promising results were seen in the use of WGA and specifically multiple displacement amplification (MDA), on forensically relevant trace DNA samples [165-167]. STR profiling success was increased significantly, although peak height biases increased genotyping difficulty. It was, however, possible to optimize the commercial MDA methods for specific application with trace DNA by using molecular crowding [167] and altered denaturation methods [168] to promote balanced amplification. Newer WGA methods are currently emerging to cater for the increased DNA requirements for next-generation sequencing and SNP genotyping, and methods are emerging that eliminate bias and non-template amplification, allowing genotyping of 550,000 SNPs from sub-nanogram amounts [169]. The continued research and development of WGA may lead to a method sufficiently reliable to allow widespread use on trace DNA samples.

### Improving detection of amplified product

The level of detection and ease of typing can be increased by manipulating the PCR product post-amplification. The most common method is to purify the PCR amplicons of the STR loci, removing salts, ions and unused dNTPs and primers from the reaction using filtration (for example, Microcon filter columns), silica gel membranes (for example, Qiagen MinElute columns), or enzymatic hydrolysis (for example, ExoSAP-IT) [79,81,170]. Purification to remove negative ions such as Cl^- ^prevents inter-molecular competition occurring during the electrokinetic injection, ensuring that the maximum amount of DNA is introduced [171]. Purification by MinElute columns gave fourfold increases in peak heights, with only small increases in the numbers and scale of artefacts [170].

During the purification process it is also possible to concentrate the PCR product, to allow even more of it to be injected. Reducing PCR product volumes from 50-25 μL to 10 μL, can produce a six to 14-fold increase in peak height [79,170]. If the entire PCR product is reduced to ~1 μL for analysis peak heights can be increased even further, although the level of artefacts significantly increased [170]. In addition, a more purified PCR product can be added to the denaturing formamide for injection - using 10 μL instead of 1.5 μL of the product gives a further increase of ~12-fold over just purification and concentration [170].

In addition to purifying and concentrating the PCR product, the amount of product injected into the capillary can be increased either by raising the time or voltage, or both, of the electrokinetic injection. Although this may not be advisable on a non-purified product (due to the higher concentration of competing ions), increasing the injection time and voltage to 15 s at 9 kV can give about nine additional alleles per STR profile for trace DNA samples and increases peak heights sixfold [172]. However, comparison with profiles generated with an increased cycle number (34 instead of 28) showed that the latter method generates the most complete profiles with the highest peak heights [172]. However, combining purification with a 30 s 4 kV injection strategy gave an increased amplification success with lower levels of stutter and peak imbalance than the standard 34-cycle methodology and can be viewed as a more flexible approach to increasing trace DNA profiling success [79]. However, there is no clear consensus on the best injection profile to use - some laboratories have validated injection strategies of 6 kV for 30 s [173], or 5 kV for 15 s [80] or different conditions dependent on the amount of DNA (1 kV for 22 s for 100 pg, 3 kV for 20 s for 25-50 pg, 6 kV for 30 s for <25 pg) [110]. From the range of views regarding the 'best' injection profile, it appears that the degree of enhancement increased injection can give is highly dependent on the STR kit and the individual capillary electrophoresis (CE) machine being used and each laboratory should, therefore, investigate its own best levels.

Newer polymers such as POP-6 and POP-7 (Applied Biosystems) have increased the sizing precision available with capillary electrophoresis, but unfortunately seem to have decreased the sensitivity of detection (KNB, data not shown). This has required further optimization of the injection parameters to achieve comparable levels of peak heights between the polymers, with a change in the recommended default injections from 3 kV for 10 s with POP-4 to 1.2 kV for 23 s with POP-7. However, the use of altered fluorophores can overcome the decreased sensitivity, as they display higher levels of fluorescence and lower quenching over time than the currently used fluorophores - FAM and ROX. Newer dyes, such as Molecular Probes' BODIPY or Alexa Fluor Series, have become commercially available, with the latter being the brightest and most photostable oligonucleotide conjugates available, with average fluorescence greatly exceeding that of even 6-FAM [174]. Energy transfer (ET) cassettes, which contain both donor and acceptor dyes separated by a sugar-phosphate spacer, give substantially higher sensitivities than single dye fluorophores [175,176]. The incorporation of these more sensitive dyes into the commercial STR amplification kits may decrease the amount of template/PCR product by lowering the limit of detection.

### Difficulties with improving amplification and typing of trace DNA

Despite all the above options for improving the amplification and detection success of trace (or, indeed, any forensic sample), many forensic laboratories have been reluctant to validate and implement them into standard case-work procedures. It is only recently that internal validations of trace DNA profiling using commercial multiplexes (with and without extended cycles/LCN methodology) have been published in peer-reviewed literature [79,80,173]. However, none of the commercial kits are validated by their manufacturer for 34 cycles or very low template amounts. The lack of validation is partially due to the innate variability that exists with trace DNA samples and their analysis. Any trace DNA sample, by its very nature, is below the stochastic threshold and is, thus, subject to the inherent variation which comes with operating at such low levels. A major cause of the reluctance in the forensic community to use methods designed for successful trace DNA analysis may be the increased level of artefacts that result from the increased sensitivity. Concomitant with the ability to amplify minute quantities of material is the increased likelihood of contamination being detected and of artefacts of the amplification process being increased due to stochastic effects. Four features are common across many trace DNA amplifications:

1. allele drop-out due to preferential amplification of one allele at one or more heterozygous loci. This is a near-constant feature of extremely low template and increases as template levels decrease [77,78,177,178]. Despite improving technologies rapidly increasing sensitivity, any amplification of trace DNA amounts must be interpreted in light of the probable drop-out of alleles

2. decreased heterozygote allele balance within a locus and between loci. The same stochastic sampling and amplification effects that cause drop-out also cause extreme peak height imbalance within and between loci, which can create difficulties evaluating zygosity at particular loci

3. allele drop-in, due to amplification artefacts such as stutter. Artefact rates can substantially increase with trace DNA amounts [78], leading to difficulties in characterizing the number of donors to a STR profile and to the assigning of alleles within a mixture

4. allele drop-in, caused by sporadic contamination occurring from either the crime-scene or the laboratory. Although the probability of allele drop-in remains the same, regardless of the template amount, the probability of detection increases with lowered genuine template levels [77,80,173]. Additionally, depending on when the contaminating source entered a sample, reanalysis may or may not reproduce the initial result.

As there are currently no methods to completely eliminate artefact product during the amplification of trace DNA, strategies are being developed to account for them statistically. However, this requires an in-depth understanding of the factors that may cause each type of artefact and accurate data regarding the frequency and scale of their occurrence, both of which are still being researched. An excellent example of the type of research required is that of Benschop *et al. *[179], which represents one of the first large-scale efforts to characterize artefacts generated by different trace DNA amplifications and typing strategies and an investigation of the most effective method to generate a useful consensus profile. The few validation studies that have been published to date [79,80,173] represent valuable resources for estimating the occurrence of the trace DNA artefacts, but it is unfortunate that few of the LCN validation studies specifically compare low template ( < 100 pg) amplifications with the standard 28 cycle to the extended 34 cycle (with, or without, product purification), with several notable exceptions [79,179]. The difference in methodologies across the various LCN and low template amplification and analytical procedures makes it difficult to compare their relative effectiveness or to build large inter-laboratory databases detailing the frequency of artefact production at low template levels.

## Low level DNA interpretation

The interpretation of trace DNA analyses is currently the most controversial aspect of its use within the medico-legal systems. In placing a profile obtained from trace amounts of biological material found at the crime-scene into context, the analyst should take into account the potential for transfer of the material, the possible cellular origin of the DNA profile in question, the stochastic nature of the collection and analytical procedures and the possibility of artefacts confounding the true result. In most laboratories the analytical methods and statistical calculations employed for standard DNA typing are used for trace DNA - a process which is statistically and scientifically incorrect and which can bias calculations heavily against the defendant. In 2007, a high-profile case in Northern Ireland [180] raised questions regarding the appropriate interpretation methods of low template DNA and the subsequent UK Forensic Regulator's report recommended the development and validation of methods specific to trace DNA amounts [181].

### Interpretation methods specific for trace DNA

Guidelines and models for the interpretation of trace level DNA profiles have existed for over a decade [77] but there has been no widespread implementation across laboratories performing low template analysis. Instead, the same profile interpretation and statistical methods are used as for high quantity samples. However, it is imperative that any analysis of a trace profile considers the four most common features of trace amplification: allele drop-out, decreased heterozygote balance, allele drop-in (stutter) and contamination, as described above. The effects of these can be minimized by the implementation of strict interpretation guidelines and specialized statistical models and can give the user reliable and robust results from trace DNA.

The most common method of ensuring the reliability of trace DNA profiles involves the use of detection thresholds. In order to eliminate background noise, a ~50 RFU threshold is commonly used as a calling threshold, termed the limit of detection (LOD). To ensure allelic drop-out does not result in false homozygote calls, a separate threshold, referred to as the low-template DNA threshold, *T *[182], the MIT (match interpretation threshold) [183], or the limit of quantitation (LOQ) [184] is set at 150-200 RFU. Only peaks above this threshold may be called as homozygous. The purpose behind this decision is to ensure that the probability of allele drop-out (Pr(*D*)) is minimized and so the probability of defining either a mixture as a single-source, or a heterozygote as a homozygote, is low. However, even with a strict threshold, drop-out may still occur. Therefore, it has been recommended that, instead of thresholds, a more continuous measure should be used which is modelled on the risk of dropout based on peak heights. In this manner, the evidence intensity can be included in the exclusion calculation and informative alleles below an arbitrary threshold value do not have to be automatically ignored [91].

The use of replicate reactions and the generation of consensus profiles (termed the 'biological model') have been advocated by many as the best way to ensure that reporting incorrect genotypes is minimised [77,78,179,185]. When the Random Man Not Excluded (RMNE) interpretation method is employed the consensus profile is considered necessary in order to account for the inherent stochastic variation in low template amplifications [77,78]. However, when allele drop-out is possible the use of the 2p rule may be non-conservative [186], and wastes information regarding peak height and dropout in the calculation [187]. In addition, there is no consensus on either the minimum number of replicates needed or how frequently one needs to observe an allele within the number of replicates conducted for it to be considered a true allele [90]. However, recent work has provided considerable empirical evidence suggesting that four replicates, with reported alleles detected at least twice, is the most accurate [179]. In particular, the practice of omitting loci which may have dropped out from the RMNE or probability of exclusion (PE) calculation method can be biased against the suspect, particularly as the probability that drop-out has occurred increases [89,188]. The International Society for Forensic Genetics (ISFG) has therefore recommended that RMNE is only used to interpret profiles where there is no probability of drop-out or increased stutter, which effectively rules out its use for trace DNA interpretation [188,189]. Some authors are advocating a move to a Bayesian based, likelihood ratio (LR) framework, where information regarding the probability of artefacts obscuring the true profile can be incorporated into the strength of evidence calculation [77,89,91,187,190]. The statistical model, as proposed by Gill *et al. *[77], and then expanded over the last decade [89,91,191], provides the necessary probabilistic methods, where the probability of observing the evidence profile can be combined with prior knowledge regarding dropout, the number of potential contributors, the possibility of contamination and many other factors. In addition, it is possible to use individual replicates within the likelihood ratio (LR) framework, rather than losing important information by generating consensus profiles. A customized expert interpretation system, LoComatioN, allows the automated evaluation of multiple genotypes and incorporates all necessary stochastic effects into the probability calculation [192]. Tvedebrink *et al. *[193] have begun the process of generating the empirical data and statistical models necessary to provide estimates of drop-out and it is likely that, in the coming years, firmer estimates will become available.

## Trace DNA mixture interpretation

Mixed trace DNA profiles add yet another level of complexity to the interpretative process. Mixed samples may be composed of one or more major contributors with high quantities of DNA and with a minor contributor present only at trace levels. Alternatively, all contributors' DNA within the mixture may be at trace levels. Furthermore, DNA truly derived from a single source could be treated as a mixture due to high stutter peaks being present and, therefore, wrongly interpreted as coming from multiple individuals. Given the high probability of drop-in, drop-out and increased stutter, estimating the number of contributors can be problematic, as can separating the contributors' genotypes at any given locus. Amplification bias may cause the minor contributor's alleles to drop out entirely at some loci or may cause over-amplification of some alleles, creating the appearance of a separate contributor. In particular, the increased stutter seen with trace DNA amplification [77,78,179] creates formidable problems for mixture interpretation. Although there are locus-specific stutter percentage guidelines for standard template amounts, none exist for trace DNA amounts. In addition, there is evidence that both forward and backward stutter increases with increasing allele length within a locus [20,194-196]. A difference in stutter percentage of 12% has been reported between alleles 10 and 17 at a commonly used locus [20]. From the limited data available, peak heights of backwards stutter may increase from 0%-4% at the smallest allele within a locus, to 12%-20% at the largest [194]. This difference may create a bias during interpretation, with longer molecular weight stutters being more likely to exceed thresholds and to be incorrectly designated as real. Alternatively, a peak at a stutter location of a small allele could potentially be perceived as a stutter when actually it represents a true allele from another source. Further efforts toward more precisely defining stutter peak expectations based on the laboratory specific methodologies in use, DNA template amount and allele height should assist profile interpretation.

The ISFG recommendations on mixture interpretation [189] advocate an LR approach for low template level mixtures and the incorporation of an assessment of the probability of allele drop-in, and drop-out, when considering a mixture. In contrast, Budowle *et al. *[183] prefer the PE calculation (equivalent to 1-RMNE), over what they view as the complicated nature of the LR approach and the difficulty of conveying the information to the court. However, any perceived difficulty should not automatically exclude the use of the LR approach, particularly when the PE method can introduce a substantial bias under certain conditions [186-188]. It is clear that more empirical, quantitative data on the effect of low template amounts within mixtures should be generated. To this end, Bright *et al. *[197] described the use of the heterozygote balance and average peak heights at each locus to calculate the mixture ratio and distinguish among the contributors' genotypes. Their work has shown that the peak height at any one locus (for the Identifiler kit) is consistent with the average mixture ratio expected, and that observed across the whole profile, to within a factor of 2 provided that the average peak height is above 267 RFU [197]. Below this height, the stochastic nature of the low template amplification renders any peak height threshold inaccurate. Therefore, caution has been urged for mixture interpretation when only trace amounts DNA of one or more of the contributors is present. Expert software systems such as TrueAllele are now using quantitative probability modelling, based on peak heights and likelihood functions, to estimate the probability of different contributor's genotypes within complex mixtures, with considerable success [198-200].

The increased discussion on the appropriate interpretation for low level DNA profiles has helped to move the field forward towards a more rigorous strategy for interpreting the evidence and presenting appropriate statistical measures to the courts. While this is undoubtedly a step in the right direction, there remains much work to be done. The incorporation into the LR framework of more criminalistic aspects of trace DNA, such as the possibility of transfer, background contamination and deposition rates, will produce more conservative estimates and help to eliminate some of the critics' concerns over the validity of trace DNA as a prosecutorial tool.

## Contamination issues

Contamination is a crucial issue in the analysis and interpretation of trace DNA. Contaminant DNA may appear as either the major or minor sample within a mixture or, alternatively, may overwhelm the target DNA completely. From a theoretical perspective, any DNA deposit that is not immediately relevant to the crime being investigated can be viewed as contamination. In this light, gross or sporadic contamination may appear at any point: (1) before the crime has been committed; (2) in the interval between the crime and securing the crime scene; (3) during the investigation of the scene; and/or (4) within the laboratory.

The first point can be viewed as the level of background DNA present under normal circumstances [88,201,202]. The second may occur as a result of innocent interactions by unrelated individuals. Although these two contamination points, termed adventitious transfer, cannot be strictly controlled, there are methods to account for and to minimize the impact of such contamination. These approaches/methods include:

1. perform more studies similar to those of Raymond *et al. *[67], Cook and Dixon [202], Dowlman *et al. *[203] and Toothman *et al. *[201] in order to learn more about the occurrence and persistence of DNA on particular surfaces in different environmental conditions

2. improve sample collection targeting (see above)

3. develop sample collection methodologies and strategies to decrease unwanted underlying DNA

4. apply cell separation techniques to mixed samples prior to DNA extraction (see above)

5. where possible obtain a reference sample for DNA profiling from the normal user(s) of an exhibit of interest and/or the person(s) who may have come into contact with it after the offence to assist profile interpretation

6. where possible obtain a profile of the background DNA from an area immediately adjacent to the target area to assist profile interpretation.

The third point of possible contamination at the crime scene can be controlled by the implementation of operating procedures designed to minimize the potential for contamination. Substantial amounts of contamination may occur from investigators moving, talking or coughing over exhibits, with the degree of contamination proportional to the distance from the exhibit [204]. At a minimum, standard operating procedures to limit contamination at a scene should include:

1. restricting access to a potential crime related exhibit or scene

2. the use of gloves and mouth masks by all those needing to touch and/or closely look at the possible crime related exhibit

3. regular changing of gloves by those touching exhibits

4. avoiding, as much as possible, touching areas on an exhibit that may be sampled for DNA

5. the availability of DNA profiles of all those individuals knowingly being in contact with an exhibit to check for potential contamination. Such data would assist in the interpretation of profiles.

An additional point to be aware of is the use of collection/detection devices on multiple exhibits and at multiple scenes. Fingerprint brushes are able to transfer amounts of DNA between exhibits that could generate profiles and may retain biological evidence for a considerable period of time [205,206]. Any device, therefore, which is in physical contact with a biological stain should be thoroughly decontaminated between use on exhibits or, preferably, DNA-free disposable equipment should be applied.

The fourth point, within laboratory contamination, may occur from individuals within the laboratory, inter-exhibit transfer or manufacturer-based consumable contamination. Although these events are generally the most strictly controlled for, high profile instances of gross contamination have been reported [207-209] and research has shown that, despite strict operating and cleaning procedures being in place, DNA may be transferred between exhibits and equipment [210]. Methods to minimize the possibility of contamination in the laboratory may include the:

1. use of DNA-free plasticware and consumables. The recent 'phantom of Heilbronn' incident in Germany and Austria, in which 'sterile' swab contamination during its manufacture caused police to link 40 crime scenes incorrectly [208]. To prevent this occurring, SWGDAM (Scientific Working Group on DNA Analysis Methods), ENFSI (European Network of Forensic Science Institutes) and BSAG (Biology Specialist Advisory Group) have now issued a position statement with specific recommendations for manufacturers and laboratories [211], to ensure that all materials used are DNA-free, rather than simply 'sterile'.

2. frequent and thorough cleaning of work areas within laboratories

3. periodic assessment of the level and location of DNA within the work place and on relevant tools to be performed and results considered from a risk management perspective. Where possible, recommendations derived from results relating to opportunities for improvement should be implemented [210]

4. separation of the work areas for item examination, DNA extraction and DNA amplification and typing

5. different exhibits being examined at different locations and/or times and the recording of where, when and by whom an exhibit was examined. This can assist in investigations of unexpected contamination events that may be detected at a later stage in analysis. The analysis of reference samples after the conclusion of crime scene samples can also aid in minimizing both inter-case contamination and the possibility of biased interpretation of the crime scene samples

6. negative controls being used at every stage of the analysis, including item examination and sampling.

Every trace DNA profile should be interpreted in the context of possible contamination. A mixed sample may contain background DNA, crime-related DNA and post-crime contamination, and it may be difficult to identify the relevant profile. In particular, the recent increase in cold-case investigations using DNA profiling increases the risk of detecting such samples, which may not have been collected, stored or examined with trace DNA detection sensitivities in mind. Thus, continued research, monitoring and a reduction of the incidence of all types of contamination is important for the further development of trace DNA analysis.

## Transfer issues

Throughout the years of trace DNA use, the major focus has been on improving techniques in order to obtain highly discriminating genetic profiles from minute amounts to help identify the person from whom the DNA at a crime scene is derived. However, much less effort has been expended on understanding the activities that explain how the DNA got there.

Although the first report on trace DNA identified the occurrence of secondary transfer [51], debate regarding the existence of secondary transfer followed for several years [212]. However, empirical research has since demonstrated substantial levels of transfer under a variety of situations, confirming that secondary, and possibly further transfer, may well be occurring in a number of casework situations [210,213-215].

Greater effort needs to be made by police/crime investigators to investigate how a DNA sample arrived at the location where it was found, as well as by scientists to better understand the impact of activities on the relative amounts of DNA from particular sources at a crime scene. In some instances, it is possible to derive the chain of events that led to a trace DNA sample being present at a crime scene - for example, prior visits to the scene or the known use of an item. Awareness of these variables, and their impact on transfer events, will assist in weighting the likelihood of proposed alternative scenarios. Some preliminary contributions to our knowledge of transfer in relation to residential burglary and street robbery have recently been made [67]. Others have also started collecting this type of data with preliminary investigations focusing on the type of biological material being deposited, the condition of the biological material, the type of substrate on which the biological material is present and with which it comes into contact and the manner of contact [216-218]. It is clear that more data on these and other variables are necessary to improve the accuracy of the likelihood assessment of alternative scenarios.

## Concluding remarks

Surveys of forensic practitioners regarding aspects of training, proficiency testing, procedures, methods, policies, contamination prevention, data collection and communication relating to forensic trace DNA have highlighted the need for improvements in these areas [135,136]. A number of recent reports have recommended the need for substantially greater investment into forensic services related research and development [82,219]. This review identifies how far we have come in the use of trace DNA in order to assist forensic investigations in recent years, but it also identifies several opportunities for improvement in most facets of trace DNA work. A deeper consideration of workflow processes and priorities may yield alternative protocols that allow the use of a greater portion of the available DNA, with greater sensitivity, thus increasing the chance of generating fuller and easier to interpret profiles. Further research will improve the utilisation and benefits of collecting and typing trace DNA in forensic investigations.

## Abbreviations

BSA: bovine serum albumin; LCN: low copy number; LR: likelihood ratio; LT DNA: low template DNA; PCR: polymerase chain reaction; PE: probability of exclusion; RMNE: random man not excluded; SNP: single nucleotide polymorphism; STR: short tandem repeats; WGA: whole genome amplification.

## Competing interests

The authors declare that they have no competing interests.

## Authors' contributions

RAHvO and KNB contributed equally to this work. All the authors wrote and approved the final manuscript.
